# Global analysis of the apple fruit microbiome: are all apples the same?

**DOI:** 10.1111/1462-2920.15469

**Published:** 2021-04-01

**Authors:** Ahmed Abdelfattah, Shiri Freilich, Rotem Bartuv, V. Yeka Zhimo, Ajay Kumar, Antonio Biasi, Shoshana Salim, Oleg Feygenberg, Erik Burchard, Christopher Dardick, Jia Liu, Awais Khan, Walid Ellouze, Shawkat Ali, Davide Spadaro, Rosario Torres, Neus Teixido, Okan Ozkaya, Andreas Buehlmann, Silvana Vero, Pedro Mondino, Gabriele Berg, Michael Wisniewski, Samir Droby

**Affiliations:** ^1^ Institute of Environmental Biotechnology Graz University of Technology Petersgasse 12 Graz 8010 Austria; ^2^ Department of Ecology, Environment and Plant Sciences Stockholm University Stockholm Sweden; ^3^ Department of Natural Resources Institute of Plant Sciences, Agricultural Research Organization, Newe Yaar Research Center P.O. Box 1021 Ramat Yishay 30095 Israel; ^4^ Department of Postharvest Science Agricultural Research Organization, The Volcani Institute P.O. Box 15159 Rishon LeZion 7505101 Israel; ^5^ The Robert H. Smith Institute of Plant Sciences and Genetics in Agriculture, Faculty of Agriculture The Hebrew University of Jerusalem Rehovot Israel; ^6^ United States Department of Agriculture Agricultural Research Service (USDA‐ARS). Appalachian Fruit Research Station Kearneysville West Virginia 25430 USA; ^7^ Chongqing Key Laboratory of Economic Plant Biotechnology, College of Landscape Architecture and Life Sciences Chongqing University of Arts and Sciences, Yongchuan Chongquing 402160 China; ^8^ Cornell University, 5 Castle Creek Drive, 112 Barton Lab Geneva New York 14456 USA; ^9^ Agriculture and Agri‐Food Canada, Research Farm Vineland Ontario Canada; ^10^ Agriculture and Agri‐Food Canada, 32 Main Street Kentville Nova Scotia B4N 1J5 Canada; ^11^ Department of Agricultural Forestry and Food Sciences (DISAFA), AGROINNOVA—Centre of Competence, University of Torino, Largo Braccini 2 Grugliasco (TO) 10095 Italy; ^12^ IRTA, Parc Científic i Tecnològic de Gardeny, Fruitcentre building Lleida Catalonia 25003 Spain; ^13^ Department of Horticulture, Faculty of Agriculture 1330 Cukurova University Adana Turkey; ^14^ Agroscope, Competence Division Plants and Plant Products, Müller‐Thurgaustr 29 Wädenswil CH‐8820 Switzerland; ^15^ Facultad de Química–UdeLaR Cátedra de Microbiología Montevideo Uruguay; ^16^ Department of Plant Protection, Faculty of Agronomy University of the Republic Garzón 780 Montevideo 12900 Uruguay; ^17^ Department of Biological Sciences Virginia Polytechnic Institute and State University 220 Ag Quad Ln Blacksburg Virginia 24061 USA

## Abstract

We present the first worldwide study on the apple (*Malus × domestica)* fruit microbiome that examines questions regarding the composition and the assembly of microbial communities on and in apple fruit. Results revealed that the composition and structure of the fungal and bacterial communities associated with apple fruit vary and are highly dependent on geographical location. The study also confirmed that the spatial variation in the fungal and bacterial composition of different fruit tissues exists at a global level. Fungal diversity varied significantly in fruit harvested in different geographical locations and suggests a potential link between location and the type and rate of postharvest diseases that develop in each country. The global core microbiome of apple fruit was represented by several beneficial microbial taxa and accounted for a large fraction of the fruit microbial community. The study provides foundational information about the apple fruit microbiome that can be utilized for the development of novel approaches for the management of fruit quality and safety, as well as for reducing losses due to the establishment and proliferation of postharvest pathogens. It also lays the groundwork for studying the complex microbial interactions that occur on apple fruit surfaces.

## Background

Developing a comprehensive understanding of the plant microbiome has identified as key for establishing a second green revolution (National Academies of Sciences E, Medicine, 2019). In this regard, the sequencing of plant and microbial genomes has provided a wealth of information for developing new opportunities for crop improvement. Plants and their microbiomes have co‐evolved as a meta‐organism and the term ‘holobiont’ has been used to describe the inseparable relationship between higher organisms and their microbial communities (Zilber‐Rosenberg and Rosenberg, 2008). A growing body of information indicates that the plant microbiome is involved in many host functions, directly or indirectly affecting host physiology, biochemistry, growth, disease resistance, stress tolerance and quality, before and after harvest (Berg *et al*., 2016). This field of research has already provided new applications with the ‘microbiome factor’ being included in breeding strategies, seed production, preharvest disease control and the management of postharvest pathogens (Berg *et al*., 2016; Gopal and Gupta, 2016; Wei and Jousset, 2017).

Domesticated apple (*Malus × domestica* Borkh.) is one of the most popular edible fruits worldwide and is the largest fruit crop produced in temperate regions. The global production of apple has more than doubled in the past 20 years, from 41 million tons in 1990 to 86 million tons in 2018, with a total trading value of 7.53 billion USD (FAOSTAT, 2020). Apples are often stored for several months and up to 1 year in cold storage in conjunction with different controlled atmosphere regimes. Preventing the proliferation and development of postharvest pathogens in storage is an important challenge for maintaining fruit quality and safety. Studying the temporal changes in the assembly and composition of microbial communities on and in fruit during storage and marketing is essential for controlling postharvest diseases and reducing losses and waste along the supply chain.

Despite the existence of approximately 7500 apple cultivars, which trace to the ancestral progenitor *Malus sieversii* (Ldb.) m. roem about one tenth of this number have global prominence (Cornille *et al*., 2012). Among these apple cultivars, ‘Gala’, a cross developed in New Zealand between ‘Kidd's Orange Red’ and ‘Golden Delicious’, represents a significant portion of global apple production. ‘Gala’ and its many sports, including ‘Royal Gala’ are grown extensively in all apple‐growing regions of the world and, thus, have major economic value (Bair, 2020).

Apple tree microbiome studies have shown, as in other tree crops, that its composition is influenced by genotype, management practices, rootstock and soil properties (Abdelfattah *et al*., 2016; Liu *et al*., 2018a; Wassermann *et al*., 2019a; Abdelfattah *et al*., 2020; Cui *et al*., 2020). The apple microbiome has been comprehensively reviewed (Whitehead *et al*., 2021). However, relatively fewer studies have been conducted, on the pre‐ and postharvest fruit microbiome (Whitehead *et al*., 2021). This is despite the fact that the use of various microbial antagonists has been pursued as an alternative to the use of synthetic chemicals to manage postharvest pathogens of apples. While postharvest biocontrol products using microbial antagonists, especially yeasts, have been commercialized, their wide sprayed use is limited due to problems with efficacy and regulatory hurdles. Other researchers have argued that a greater understanding of the fruit microbiome is needed to elucidate the factors involved in biocontrol systems and that this would facilitate the development of improved strategies that rely on the use of antagonistic microorganisms for managing postharvest diseases, and perhaps physiological disorders, that occur during the marketing and long‐term storage of fruit crops (Whitehead *et al*., 2021; Droby and Wisniewski, 2018; Abdelfattah *et al*., 2018a; Angeli *et al*., 2019; Kusstatscher *et al*., 2020).

Recent studies have shown that different apple fruit tissues (calyx‐end, stem‐end, peel and mesocarp) harbour distinctly different fungal and bacterial communities that vary in diversity and abundance (Whitehead *et al*., 2021; Abdelfattah *et al*., 2016; Wassermann *et al*., 2019a; Abdelfattah *et al*., 2020). Those studies differed in several respects, although the same general patterns were observed. The question remains, however, whether the observed patterns of abundance and diversity in the different tissue‐types is generally true at a global level, despite differences in climates, management practices and cultivars. One objective of the current study was to begin to examine this question. *Malus × domestica* and its derived cultivars have common ancestors (*Malus sieversii*, *M. sylvestris, M. orientalis and M. prunifolia* that represent the primary progenitors of the modern apple (Coart *et al*., 2006). Hologenome theory suggests that hosts and their microbiomes have co‐evolved (Zilber‐Rosenberg and Rosenberg, 2008). Therefore, we hypothesized that the fruit of a commercial cultivar, such as ‘Royal Gala’, would share a ‘core’ microbiome, regardless of the global location where the fruit is produced. We also hypothesized, that the high level of genetic diversity that exists in apple and its long history of domestication may have impacted the overall composition of the fruit microbiome in a regional or local manner. Additionally, biotic and abiotic conditions and management practices may have played an important role in influencing microbial community assemblages as apple production spread from its original site of origin and domestication.

Determining the existence of a ‘core’ microbiome would provide important information on its impact on disease susceptibility and resistance and human health, as well as provide a more comprehensive understanding of fruit biology in light of the holobiont concept. A deeper understanding of the interactions between hosts and their resident microflora and how they are impacted by intrinsic (genetic) and extrinsic (management practices and the environment) can be used to develop novel approaches for the management of fruit quality, pre‐and postharvest disease and food safety. The main objectives of the present study were to determine:(i) if the spatial differences in microbial composition previously reported exist on a global scale, irrespective of where the fruit is grown; (ii) how the structure of the fruit microbiome is affected by geographical location and general differences in climate and (iii) if a core microbiome could be identified and if so how do the members of the core microbiome interact as a network. Results of the study provide a global perspective on the microbiome of apple fruit and provide a foundation for developing a better understanding of the interactions that potentially occur within the fruit microbial community, as well as the potential interactions that may occur between the fruit and its resident microflora in relation to postharvest diseases, fruit quality and food safety.

## Results

### Microbial diversity associated with Royal Gala apple

**Fig 1:**
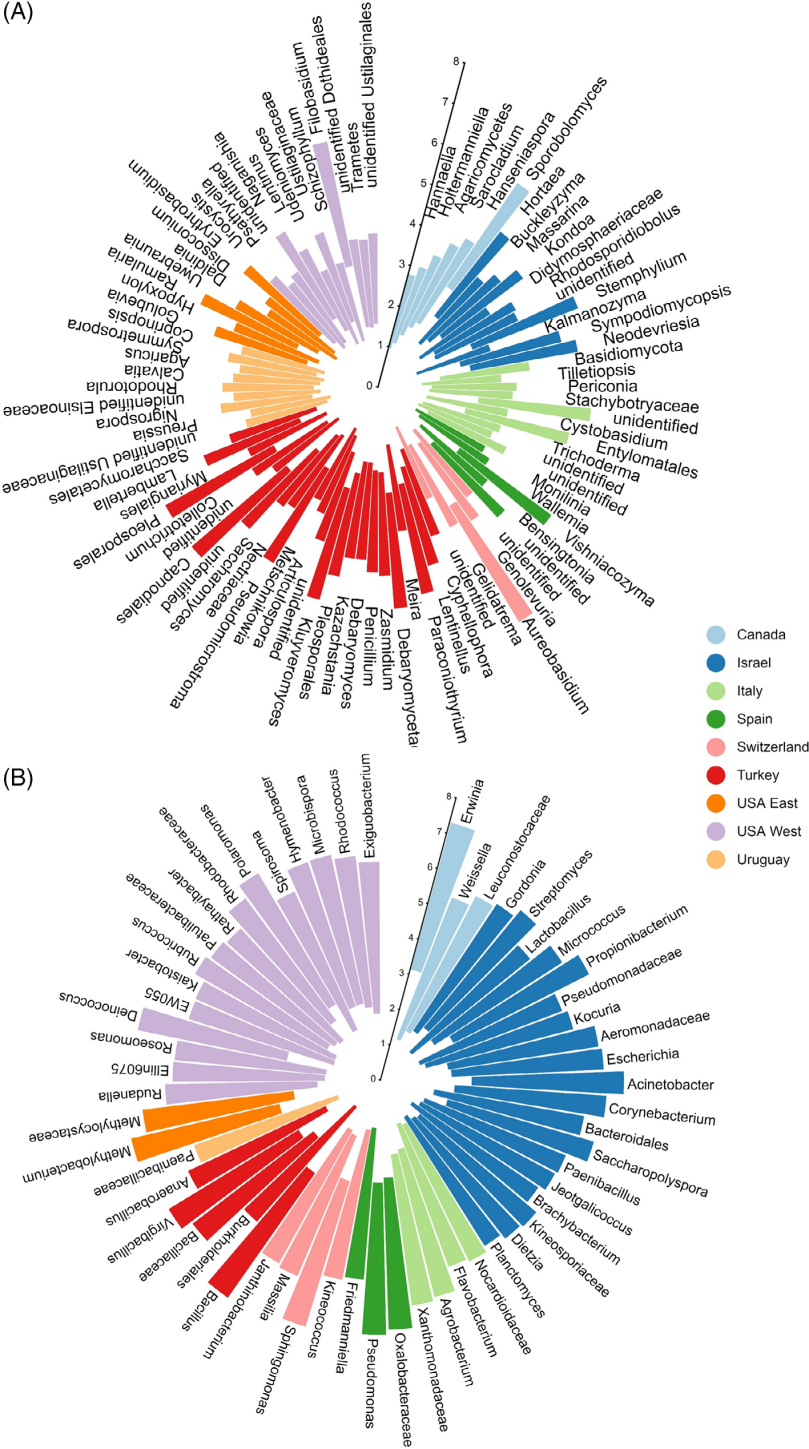
Circular bar plot of the LDA scores showing a list of (A) fungal and (B) bacterial taxa that best characterize each geographical location i.e. Canada, Turkey, Israel, Italy, Uruguay, USA West, USA East, Switzerland and Spain. Higher LDA score indicate higher consistency of differences in relative abundance between taxa of each country.

After removal of low‐quality sequences and plant sequences, 6.117.315 16S and 48.528.735 ITS2 reads were obtained and assigned to 20.072 bacterial and 16.241 fungal ASVs, respectively. The ASVs corresponded to 25 bacterial and six fungal phyla, which in turn were assigned to 558 bacterial and 822 fungal genera. The apple fungal community across the investigated countries was dominated by *Ascomycota* (79.8%) and *Basidiomycota* (9.3%), although, other phyla such as *Chytridiomycota, Entomophthoromycota, Mortierellomycota* and *Mucoromycota* were also detected at a lower relative abundance (data not shown). Within the Ascomycota, genera such as *Aureobasidium* (29.00%), *Cladosporium* (16.60%) and unidentified groups of *Capnodiales* (8.80%) and *Pleosporaceae* (8.50%) represented more than 60% of the total fungal community (Table S2). *Sporobolomyces* (5.70%), *Filobasidium* (4.20%) and *Vishniacozyma* (1.60%) were the predominant *Basidomycota*. Regarding bacteria, *Proteobacteria* (65.1%), *Firmicutes* (15.8%)*, Actinobacteria* (15.1%) and *Bacteroidetes* (2.3%) were the most prevalent bacterial phyla, representing 98.3% of the entire bacterial community. The abundance distribution of the bacterial phyla was consistent across countries, except in Turkey where *Firmicutes* were more abundant than *Proteobacteria* compared with the other countries. *Sphingomonas* (12.40%), *Erwinia* (11.30%), *Pseudomonas* (9.30%), *Bacillus* (7.10%), unidentified *Oxalobacteraceae* (6.80%), *Methylobacterium* (6.20%) and unidentified *Microbacteriaceae* (5.90%) were the most abundant bacterial genera. (Table S2). Results of the linear discriminant analysis (LEfSe) revealed 90 fungal and 57 bacterial taxa characterized each of the investigated countries (Fig. 1). Samples from Turkey had the highest number of fungal genera (25), which included *Penicillium*, *Zasmidium* and *Pseudomicrostroma*. In contrast, samples from Spain had the lowest number of fungal genera (5), which included *Monilinia, Vishniacozyma and Bensingtonia* (Fig. 1A). Samples from Israel and the western USA had the highest number of unique bacterial taxa, while only one bacterial taxon, identified as within the *Paenibacillaceae* was observed in samples collected in Uruguay (Fig. 1B).

### The effect of growing region on the microbial diversity of apple fruit

**Fig 2:**
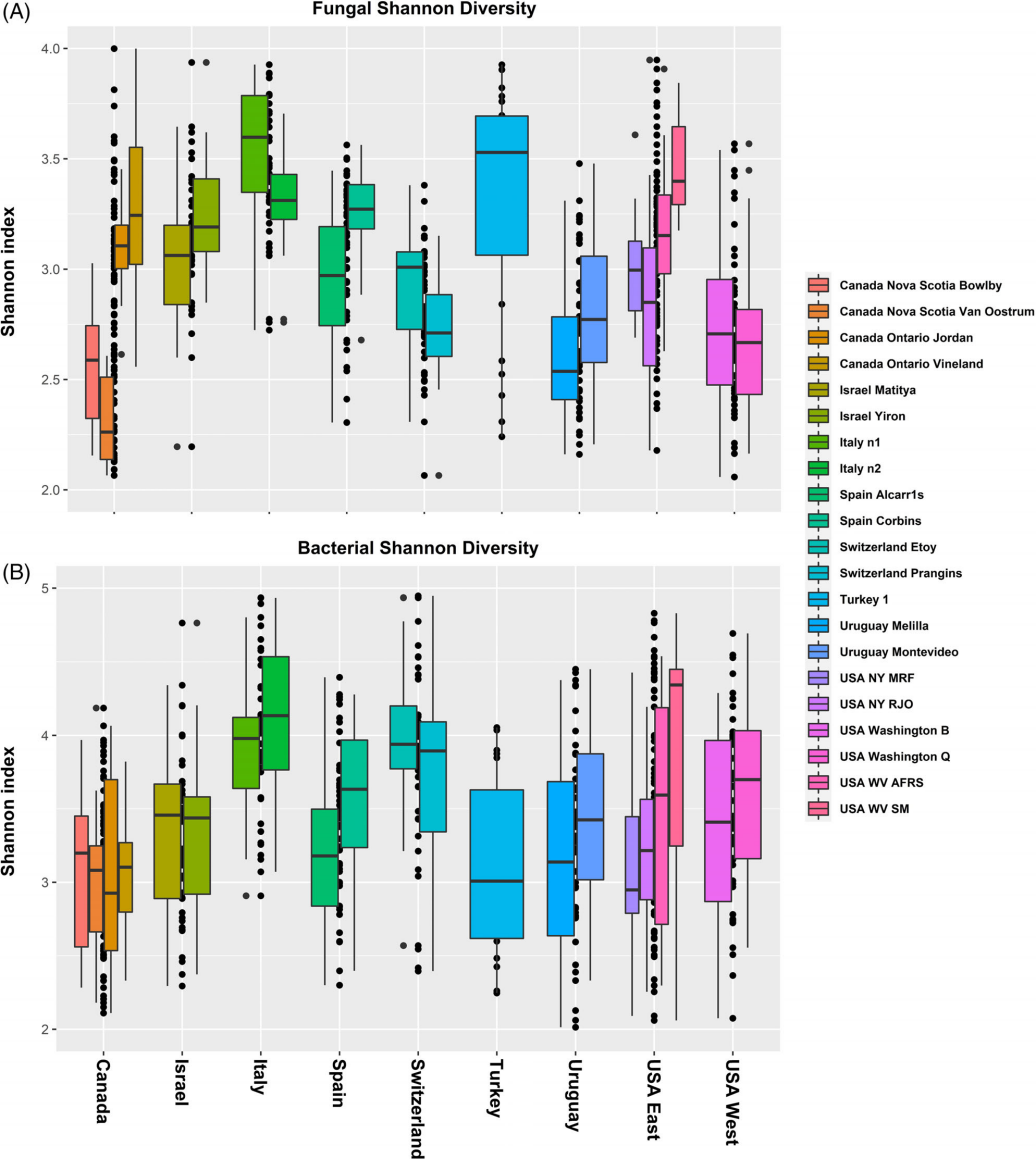
Box plots showing the bacterial fungal diversity (Shannon index) of apple samples collected from different countries (Canada, Turkey, Israel, Italy, Uruguay, USA West, USA East, Switzerland and Spain).

The geographical location in which apples were sampled had a significant effect on the microbial diversity associated with the fruit (Table 1). For example, country of origin (including location within a country) had a significant effect on the diversity of fungi (*P* < 2 × 10^−16^) and bacteria (*P* > 2 × 10^−16^). Similarly, although to a lesser extent, the effect of orchard on fungi (*P* < 2 × 10^−16^) and bacteria (*P =* 1.09 × 10^−8^) was also statistically significantly. In addition, the two‐way interactions between country and tissue type as well as orchard and tissue type were significant for both fungal and bacterial diversity. Pairwise comparison between Shannon diversity of the investigated countries indicated that both fungal and bacterial diversity differed significantly between locations and orchards within a location (Table S3). Samples from Italy had the highest fungal diversity, followed by samples from Turkey and Israel (Fig. 2A). The highest bacterial diversity was observed in apples collected from Italy, the USA and Switzerland (Fig. 2B).

**Table 1:** Analysis of Variance Model results on the effects of geographical location (country), orchard and fruit tissue type, and their interactions on Shannon diversity of bacteria and fungi on apple fruits.

	Shannon	*df*	Sum Sq	Mean Sq	*F* value	Pr(>F)
Fungi	Country	8	26.98	3.372	44.06	<2 × 10^−16^
	Orchard	12	28	2.334	30.49	<2 × 10^−16^
	Tissue type	2	0.38	0.188	2.45	0.0875
	Country × Tissue type	16	14.62	0.913	11.93	< 2e‐16
	Orchard × Tissue type	24	4.81	0.201	2.62	6.09 × 10^−5^
	Residuals	428	32.76	0.077		
Bacteria	Country	8	46.09	5.761	22.993	<2 × 10^−16^
	Orchard	12	16.51	1.376	5.491	1.09 × 10^−8^
	Tissue type	2	34.47	17.236	68.794	< 2 × 10^−16^
	Country × Tissue type	16	18.96	1.185	4.729	8.22 × 10^−9^
	Orchard × Tissue type	24	22.11	0.921	3.678	3.03 × 10^−8^
	Residuals	399	99.97	0.251		

#### Community composition of apple across countries

**Fig 3:**
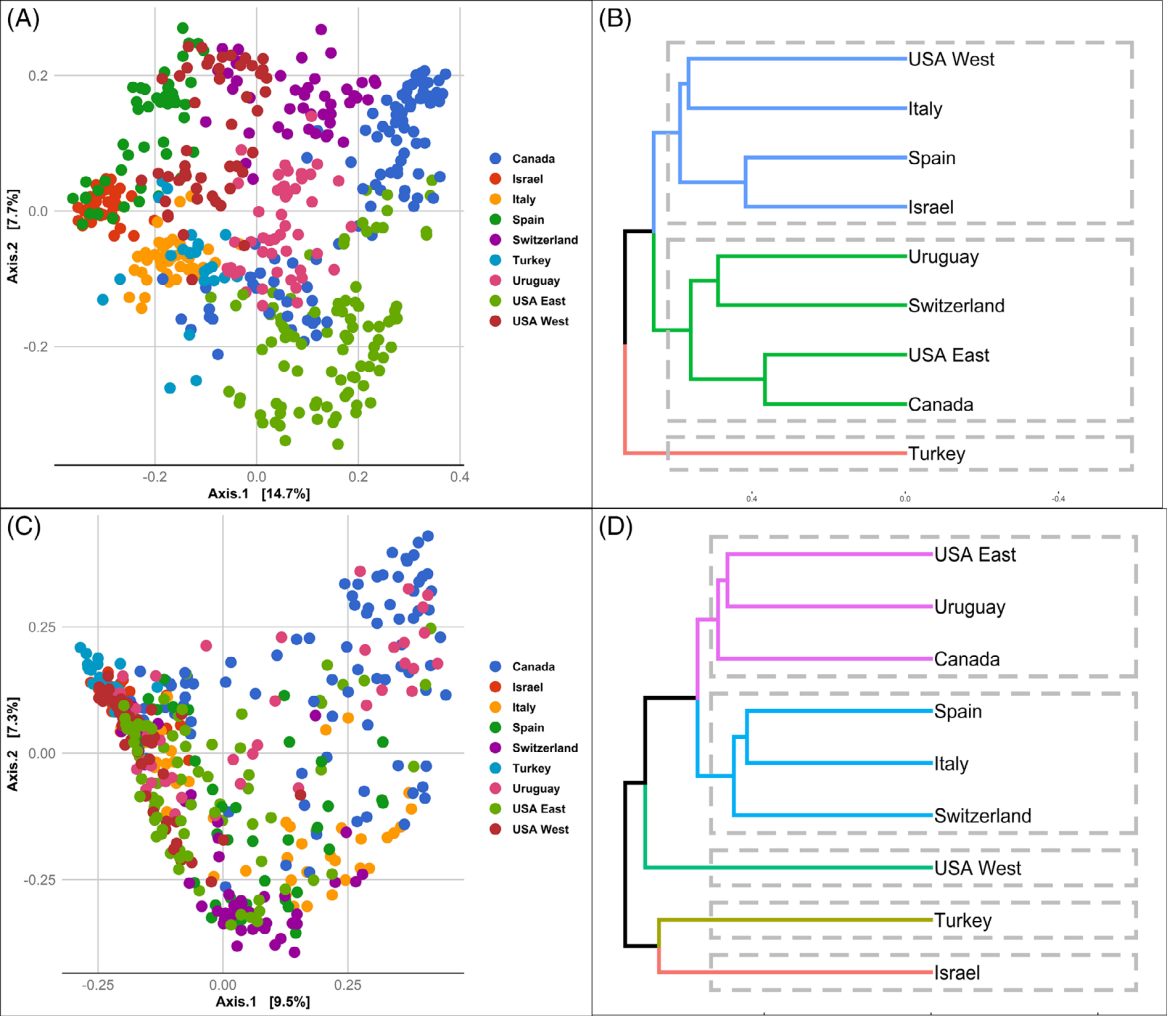
PCoA plots of the fungal (A) and bacterial (C) community compositions based on Bray–Curtis dissimilarity distances. Dendrogram of hierarchical clustering showing the similarity between apple fungal (B) and bacterial (D) communities collected from different countries i.e. Canada, Turkey, Israel, Italy, Uruguay, USA West, USA East, Switzerland and Spain. The hierarchical clustering was based Bray Curtis dissimilarity metric using ‘average clustering UPGMA’ and k mean = 4 as implemented in vegan R, where branches colours correspond to clusters.

The geographical location of the investigated sites had a significant effect on shaping the community composition of the tested apples. While the ‘country effect’ had a significant impact on the overall apple microbiome (*P* = 0.001), it explained a higher proportion of the variance in the fungal community (*R* ^*2*^ *=* 0.375) than in the bacterial community (*R* ^*2*^ = 0.152). This was also evident in the PCoA analysis based on Bray Curtis dissimilarity test (Fig. 3A and C). An effect of orchard was also observed, explaining less variation, however, in fungal (*R* ^*2*^ = 0.136, *P =* 0.001) and bacterial (*R* ^*2*^ = 0.048, *P =* 0.001) communities relative to country (Table 2). The two‐way interactions between country and tissue type as well as orchard and tissue type were significant for both fungal and bacterial communities.

**Table 2:** PERMANOVA results on testing the effect of country, orchard, tissue and their interactions on bacterial and fungal communities of apple fruits.

		*df*	Sums of Sqs	Mean Sqs	F. Model	*R* ^2^	Pr(>F)
Fungi	Country	8	48.079	6.0098	59.229	0.37528	0.001
	Orchard	12	17.478	1.4565	14.354	0.13643	0.001
	Tissue type	2	3.408	1.7038	16.792	0.0266	0.001
	Country × Tissue type	16	8.892	0.5558	5.477	0.06941	0.001
	Orchard × Tissue type	24	6.829	0.2845	2.804	0.0533	0.001
	Residuals	428	43.428	0.1015		0.33898	
	Total	490	128.113			1	
Bacteria	Country	8	30.649	3.8311	12.5466	0.15272	0.001
	Orchard	12	9.741	0.8117	2.6583	0.04853	0.001
	Tissue type	2	10.419	5.2093	17.0602	0.05191	0.001
	Country × Tissue type	16	16.21	1.0131	3.3178	0.08077	0.001
	Orchard × Tissue type	24	11.841	0.4934	1.6157	0.059	0.001
	Residuals	399	121.835	0.3054		0.60707	
	Total	461	200.694			1	

The comparisons were based on Bray Curtis dissimilarity, and *P*‐values were calculated using the adonis function in vegan and corrected using the FDR method.

Hierarchal clustering revealed that European apples (Switzerland, Italy and Spain) had a bacterial community that was more similar to each other, relative to sites in eastern North America and South America (eastern USA, Canada and Uruguay) which formed a separate cluster (Fig. 3D). Turkish and Israeli apples appeared to harbour a distinct bacterial community. Hierarchal clustering of the fungal community composition revealed that apples collected from the western USA, Italy, Spain and Israel formed a separate cluster from a cluster formed by the eastern USA, Canada, Uruguay and Switzerland (Fig. 3B). Furthermore, orchards within the same country appeared to have similar microbial communities than those sampled from another country. These results were more evident, however, in fungal communities than in bacterial communities (Fig. S1).

### Spatial variation in the apple microbiome

**Fig 4:**
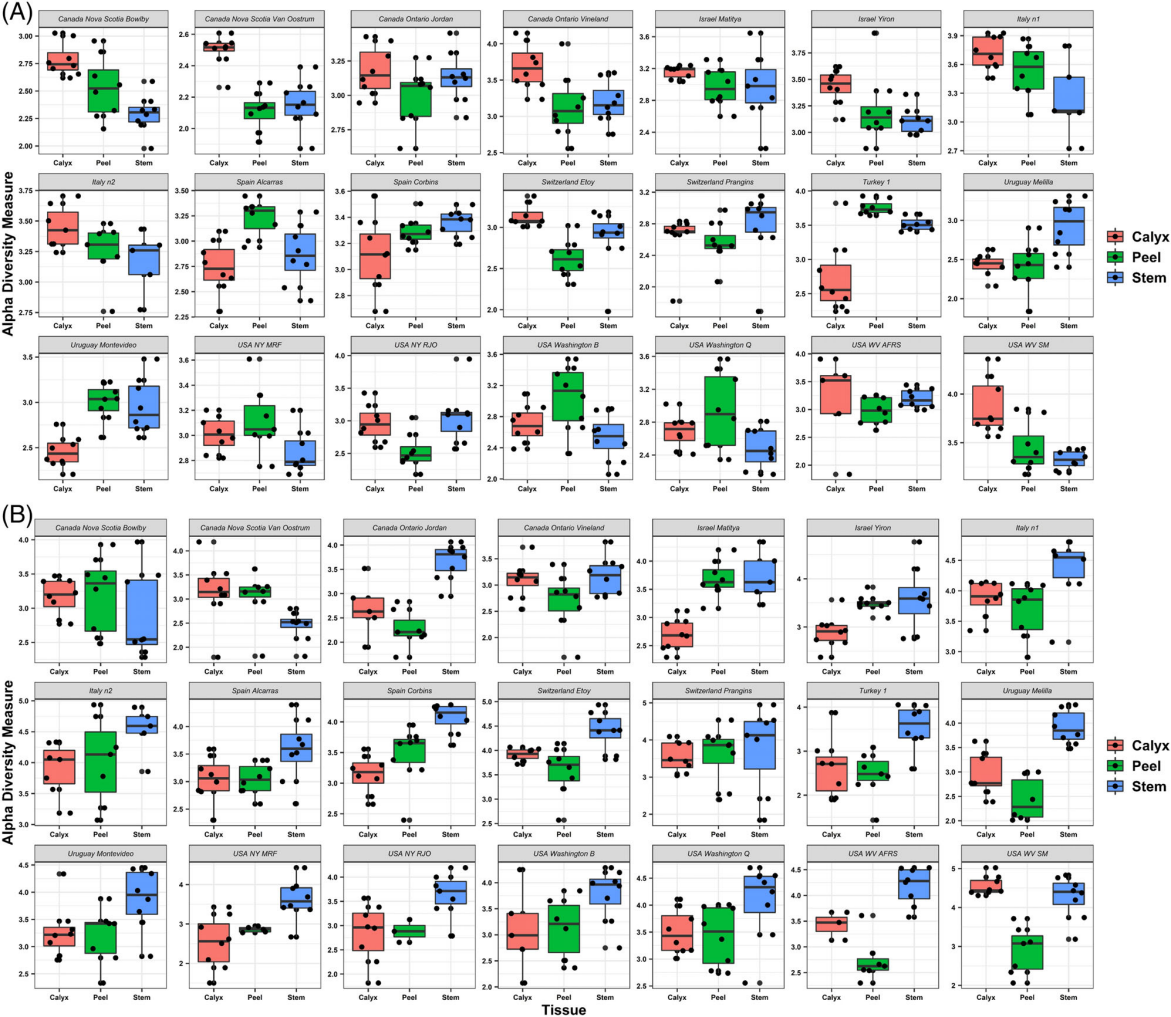
Boxplots of fungal (top) and bacterial (bottom) Shannon diversity among apple tissues (Calyx, stem and peel) collected from 21 orchards located in eight countries. The figure is arranged in seven columns and three rows for fungi (A) and bacteria (B). Each subpanel corresponds to an orchard. From left to right, the first row shows the results of Nova Scotia Bowlby (Canada), Nova Scotia Van Oostrum (Canada), Ontario Jordan (Canada), Ontario Vineland (Canada), Matitya (Israel), Yiron (Israel) and orchard n1 (Italy). The second row shows the results from orchard n2 (Italy), Corbins (Spain), Alcarràs (Spain), Prangins (Switzerland), Etoy (Switzerland), orchard 1 (Turkey), Melilla (Uruguay). The third row shows the results from Montevideo (Uruguay), MRF (NY, USA), RJO (NY, USA), orchard B (Washington USA), orchard Q (Washington USA), AFRS (WV USA), SM (WV USA). A and B are arranged in the same order.

**Fig 5:**
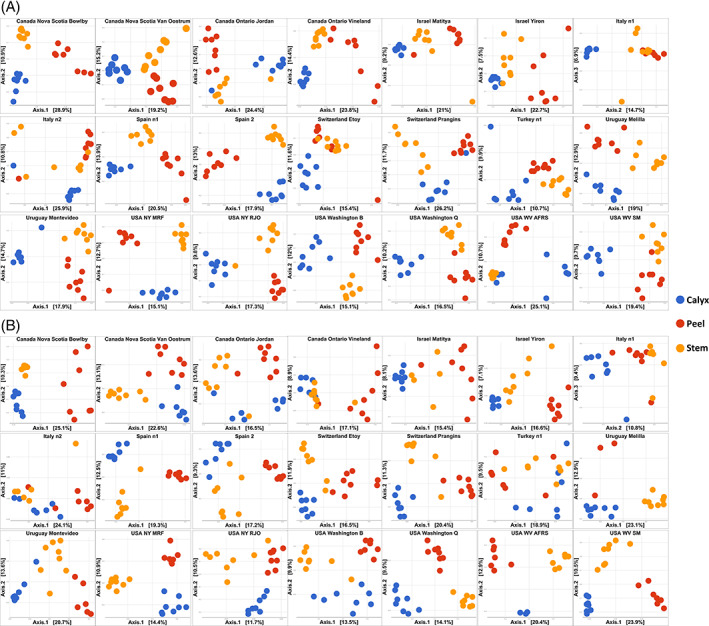
PCoA plots showing the variation in fungal (A) and bacterial (B) community composition among apple tissue types (Calyx, stem and peel). Analysis were based on Bray Curtis dissimilarity metric of CSS normalized OTU table. The figure is arranged in seven columns and three rows for fungi (A) and bacteria (B). Each subpanel corresponds to an orchard. From left to right, the first row shows the results of Nova Scotia Bowlby (Canada), Nova Scotia Van Oostrum (Canada), Ontario Jordan (Canada), Ontario Vineland (Canada), Matitya (Israel), Yiron (Israel) and orchard n1 (Italy). The second row shows the results from orchard n2 (Italy), Corbins (Spain), Alcarràs (Spain), Prangins (Switzerland), Etoy (Switzerland), orchard 1 (Turkey), Melilla (Uruguay). The third row shows the results from Montevideo (Uruguay), MRF (NY, USA), RJO (NY, USA), orchard B (Washington USA), orchard Q (Washington USA), AFRS (WV USA), SM (WV USA). A and B are arranged in the same order.

The effect of tissue types on fungal diversity (Shannon) was not statistically significant when tissue samples from all countries were grouped together (*F =* 2.45, *P =* 0.0875). The interaction between country and tissue type, as well as between orchard and tissue type, however, were significant (Table 1). In the majority of the orchards, calyx‐end tissue exhibited a higher fungal diversity, followed by peel and stem‐end tissues, with a few exceptions observed in samples collected from Uruguay, Turkey and Spain (Fig. 4A). In contrast, tissue type had a significant effect on bacterial diversity, regardless of the sampling location (*F =* 68.794, *P =* 2 × 10^−16^), as well as in the interaction between country and tissue, as well as orchard and tissue (Table 1). Stem‐end tissues harboured the highest bacterial diversity relative to fruit peel and calyx‐end tissues, except in the New Brunswick, Canada samples (Fig. 4B). PERMANOVA analysis indicated that tissue type, as well as the interaction between tissue type and country, and tissue type and orchard, had a significant effect on fungal community composition (Table 2). This effect was observed in all of the investigated orchards in all countries, based on the results of the PCoA analysis where samples collected from apple calyx‐end, stem‐end and peel, tissues clustered separately from each other (Fig. 5A). Similar results were also found for the bacterial community which differed significantly in all orchards, (Fig. 5B).

### The core microbiome of Royal Gala apple

The global core of the apple microbiome, defined at taxa present in at least 75% of the samples, consisted of six fungal genera, namely: *Aureobasidium, Cladosporium, Alternaria, Filobasidium, Vishniacozyma* and *Sporobolomyces* and two bacterial genera namely: *Sphingomonas* and *Methylobacterium*. While none of the bacterial genera was found to be prevalent in 90% of the samples, the fungal genera *Aureobasidium* and *Cladosporium* were found in up to 96% of the samples. Interestingly, the community composition of *Sphingomonas* was sufficient to distinguish between most of the investigated countries and showed niche specialization within the fruit i.e. stem‐end, calyx‐end and peel tissues harboured different Sphingomonas communities (Fig. S2). Similar results were also observed for *Aureobasidium*, a core fungal genus, however, species variability was limited, and differences were attributed to niche specialization in the different tissue‐types (data not shown).

**Fig 6:**
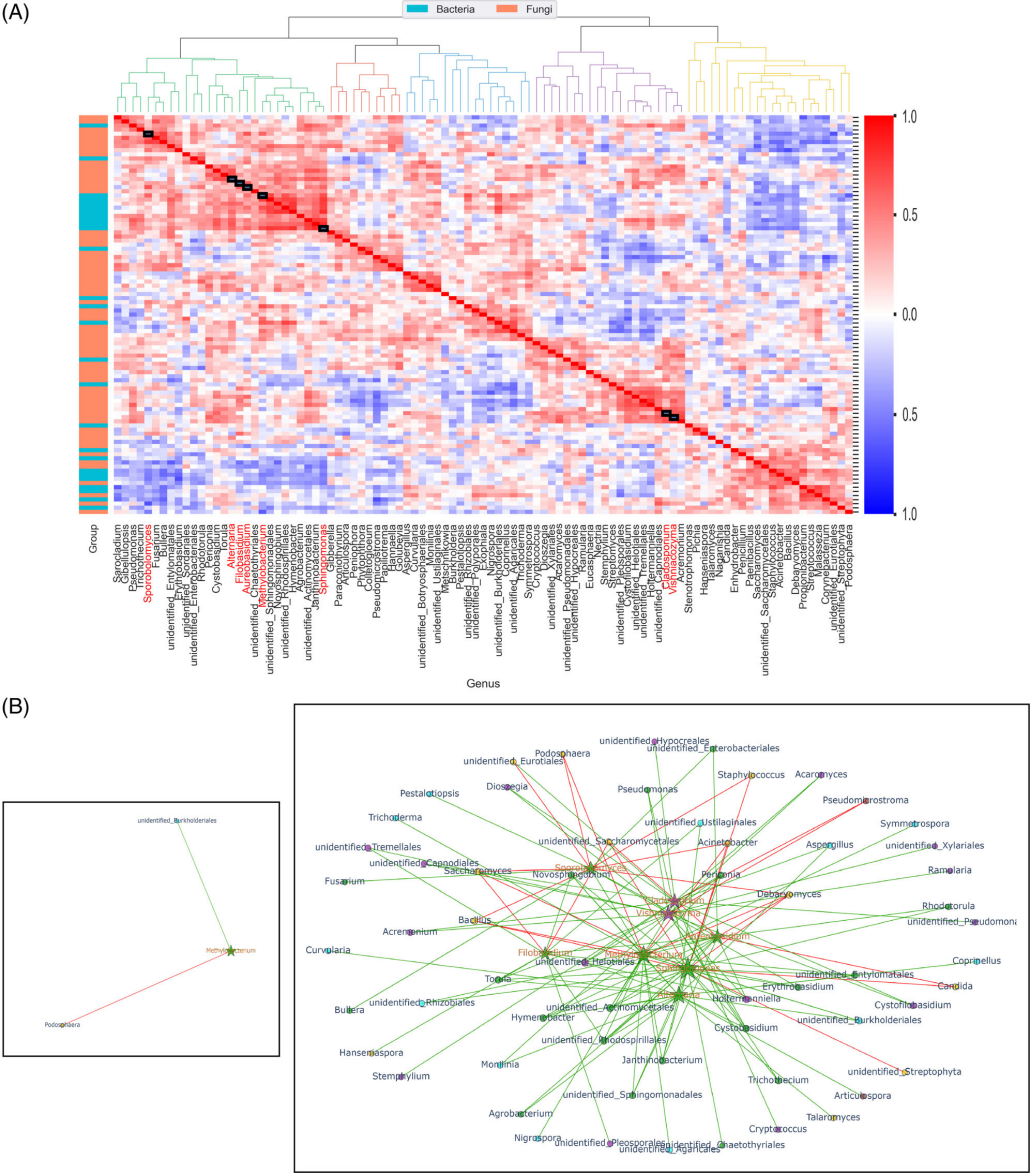
Correlation matrix (based on Spearman's rank correlation coefficient) of the abundance profiles of core and non‐core genera from the apple fruit microbiome (27 bacterial and 70 fungal species). ‘Average’ linkage was used for the hierarchical clustering. Dendrogram was divided into five groups by cutting the tree at *h* = 0.8. Black squares on the diagonal line indicate core species also labelled in red font (A). Co‐occurrence network presenting interactions involving core species. Core and non‐core species are represented by star and circle‐shaped nodes, respectively. Green and red lines (i.e., edges) represent significant positive (*r* > 0.4, *P* < 0.01) and negative (*r* < 0.4, *P* < 0.01) correlation between two nodes, respectively. The size of each node is proportional to nodes' degree (the number of edges associated with the node). Colours are corresponding to the five key clusters in panel. The black frame highlights positive and negative interaction between a core bacteria (Methylobacterium) and potential biocontrol agent (*Burkholderiales*) and pathogen, respectively (*Podosphaera*) (B). An interactive version of the network is available in Data S4.

In order to detect potential interactions between core and non‐core groups we depicted co‐occurrences by constructing a correlation matrix based on normalized distribution patterns of bacterial and fungal genera (Fig. 6A). Clustering pattern indicates that genera can be divided into five key groups of co‐occurring species mixing bacterial and fungal genera. Core species are distributed in two clusters, each hosting one of the two most dominant Ascomycota genera ‐ *Aureobasidium* (green) and *Cladosporium* (purple). Microbiomes with a high relative abundance of *Aureobasidium* and a low abundance of Cladosporium were characterized in Switzerland, USA and Canada; alternatively, high numbers of *Cladosporium* and low numbers of *Aureobasidium* were described in Israel and Turkey (Fig. S1). Considering the significant negative and positive interactions between genera, core species were found to have a significantly higher number of interactions in comparison to non‐core species with an average node degree of 19.125 neighbours in comparison to 12.23 in none core species (Fig. S2). A network formed by the interactions of core genera with core and non‐core groups is consistent of 142 edges and connects 8 and 60 core and non‐core genera, respectively (Fig. 6B). The highest number of interactions −30– was recorded for one core genus – *Sphingomonas*. Using the network, we could identify potentially useful relationships among and between core and non‐core genera within the microbial community (Fig. 6B). For example, the core genera *Methylobacterium* is positively associated with *Burkholderiales*—a group that includes reported biocontrol agents (Angeli *et al*., 2019), and a negative association with a known apple pathogen *Podosphaera*. These co‐occurrence associations can be indicative of cooperative and competitive interactions, respectively, and can serve the design of experiments to assess these interactions *in vitro* and on the fruit.

## Discussion

This is the first study to provide a global analysis of the apple fruit (‘Royal Gala’) microbiome and determine the structure and diversity of microbial communities on and in different fruit tissues at harvest. A core microbiome shared between apple samples in all locations was identified suggesting that the members of the core microbiome may have co‐evolved with the domestication of apple and potentially may play an essential role in defining fruit traits related to disease resistance and fruit quality. We characterized the microbial communities associated with ‘Royal Gala’ apple fruit at harvest maturity stage and assessed the effect of geographical location on both large‐scale spatial variations, i.e. across different countries and small‐scale spatial variations, i.e. within a fruit. While the microbiome associated with plants has been extensively studied, knowledge about the fruit microbiome is still rather limited relative to rhizosphere, endophyte and phyllosphere studies (Whitehead *et al*., 2021; Kusstatscher *et al*., 2020). Additionally, information about the role of the fruit microbiome on pre‐ and postharvest diseases, as well as fruit physiology, is also lacking. This is despite the importance of postharvest losses in reducing the economic return from fruit production, especially after so many resources have already been expended to produce a harvestable crop. Apples also encounter losses in storage, transit, markets and homes due to postharvest pathogens (Lipinski *et al*., 2013). For over 30 years, there has been considerable research focus on the development of biological control strategies based on naturally occurring microorganisms (Droby *et al*., 2016; Droby and Wisniewski, 2018). Especially with the use of yeast antagonists, has been an active area of research. Several postharvest biocontrol products based on single antagonists have been developed and registered. The large scale commercial use of these products has been limited a due to inconsistent performance under commercial conditions (Wisniewski *et al*., 2016). In this regard, Droby and colleagues 2018 have indicated that a new paradigm is needed for postharvest biocontrol to achieve commercial success and that understanding the naturally occurring microbiome of fruit surfaces and its function, will lead to the development of new biological strategies for postharvest disease control. Several studies have reported on the population dynamics of biocontrol agents on intact and wounded fruit over the course of low‐temperature storage. A wide array of mechanisms has also been demonstrated for postharvest biocontrol agents that involve yeast antagonist, the pathogen and the host. This study and others are providing the foundation for understanding the structure and function of the carposphere microbiome. Such information is an essential step towards the development of effective biological approaches to postharvest disease management. For example, efforts to modulate the gut microbiome for improved human health have moved from simple inoculations with beneficial bacteria (probiotics) to supplements that contain specific metabolites that provide a resource that can be selectively utilized by beneficial bacteria (prebiotics) to combinations of probiotics and prebiotics (synbiotics) that can more effectively shift the composition of an existing host community (Sanders *et al*., 2019). Similarly, in the apple rhizosphere, efforts to manipulate the soil microbiome to treat apple replant disease have shown that directed changes to the resource environment (e.g., through selective soil amendments) are more successful at controlling disease than inoculations with single strains or simple consortia of beneficial microbes (Raaijmakers and Mazzola, 2016; Mazzola and Freilich, 2017; Winkelmann *et al*., 2018). Research designed to identify, quantify and elucidate the metabolic networks constructed by microbial populations on harvested fruit is a fundamental need. Such studies will improve our understanding of the mechanisms that regulate the assembly of beneficial microbial communities, and lead to the development of strategies for beneficially manipulating microbial communities *in situ*.

### Geographical location

Apples represent a major item of export and are shipped globally. Therefore, it is of importance to determine if the structure of the apple fruit microbiome is relatively uniform regardless of where the fruit is produced. Rather than the presence of a uniform microbiome, the present study revealed that geographical location is a principle factor determining the structure of the apple fruit microbiome. Fungal communities, however, were more affected by geographical location (country and site within a country) than bacterial communities. The stability of the fruit‐associated bacterial community, relative to their fungal counterparts, has been previously reported in stored apples (Wassermann *et al*., 2019b; Abdelfattah *et al*., 2020). The higher level of variation in the fungal community may be potentially attributed to the fact that fungal assemblages appear to be derived from regional fungal pools with limited dispersal capability (Lumibao *et al*., 2019). In addition, we observed that as the variation in the microbial communities among sites was positively correlated with the distance between those locations, especially for fungi. For example, variations in fungal and bacterial communities associated with apple fruit were lower at a local scale, i.e., among orchards within the same geographical location, sites within a country, e.g., eastern and western USA and Canada and increased at the country level. Furthermore, a continental pattern can be drawn especially for the bacterial community which in one hand indicates adaptation of the apple microbiome to local environments, and on the other hand, it may be explained by the metacommunity theory. A metacommunity is defined as a set of local communities that are linked by dispersal of multiple potentially interacting species (Leibold *et al*., 2004). However, the present study had an insufficient distribution of samples to evaluate this premise. Nevertheless, the geographical location has been previously reported to be one of the most important determinants of the structure of the plant microbiome (Mezzasalma *et al*., 2018; Lin *et al*., 2020). A study of the maize rhizosphere found that location had a higher impact on the plant microbiome than genotype (Peiffer *et al*., 2013). Similarly, a study of the global citrus rhizosphere microbiome reported large variations in community structure that were attributed to geographical location (samples collected in different countries; Xu *et al*., 2018). The large‐scale variations between countries, together with the similarity observed among apple microbial communities within a country or region within a country, suggests that the structure of the microbial community on apple fruit is locally adapted to local environmental conditions that influence microbial diversity and composition (Hoostal *et al*., 2008). In this regard, it is also commonly recognized that the humid, wet conditions present in the eastern portions of the USA and Canada, present a much greater disease and pest challenge than the dry conditions present in the western USA and Canada. This is especially supported by the differences in diversity levels between these two contrasting environments, although more evident for the fungal community (e.g., Figure 3).

### Tissue type

Plants tissues provide a variety of niches that can harbour distinct microbial communities. Plant roots, leaves, flowers, fruit as well as other organs, represent different microhabitats, each with specific features that favour the growth of specific microorganisms in these organs. Different tissue types within the same organ have been previously reported to exhibit spatial variations in microbial community structure. For example, the upper and lower leaf sides, as well as the peel and pulp of various fruits, including apple, have been reported to exhibit differences in microbial community structure (Abdelfattah *et al*., 2016; Vionnet *et al*., 2018; Abdelfattah *et al*., 2020; Piombo *et al*., 2020). The experimental design used in the present study was selected to determine if spatial variations within a fruit are global, i.e., will be evident regardless of geographical location and the variety of environmental conditions present in the different sites. Results indicated that the effect of fruit tissue‐type on the composition of the microbial community was rather limited, *R* ^2^ = 0.0266 for fungi and *R* ^2^ = 0.05191 for bacteria, yet significant, i.e., *P* = 0.001. A larger effect was observed, however, when individual orchards were analysed separately (Figs 5 and 6). Spatial variations in fungal and bacterial community composition and Shannon diversity due to tissue‐type was consistently observed in all of the investigated orchards. These results, along with previous studies, confirms that spatial variation in the structure of the microbial community exist between tissue‐types (calyx‐end, stem‐end and peel) at a global level. Since geographical location, is the main factor shaping the structure of the apple microbiome, however, the effect of tissue‐type is greatly reduced when samples of tissue‐types are pooled across countries. Notably, the association of a distinct microbiome with such a small environmental niche (tissue‐type) suggests specialized adaptation and function to those microhabitats. We suggest that the conditions (morphological, nutrient and environmental) present in each of these microhabitats (tissue‐types) could play an important role in determining community structure. For instance, the calyx‐end is an open site that may create special niche for specialized fungi such as *Alternaria* and other fungal pathogens which can cause internal rots. Interestingly, *Erwinia* species were found at higher abundance in the Calyx‐end tissue compared with the other tissue types, especially in Canadian apples. This can be explained by the fact that the calyx contains floral residues which are most affected by *Erwinia amylvora*, the cause of fire blight disease of pome fruit.

### Core microbiome

A core microbiome is a set of microbes consistently present over time on a specific host and is likely to be critical to host development, health and functioning (Berg *et al*., 2020). Defining the core microbiome enables researchers to filter out transient associations and focus on stable taxa with a greater likelihood of influencing host phenotype and is therefore essential in exploring the potential for pre/probiotic treatments that support host health (Berg *et al*., 2020). In this study, the core microbiome of apple fruit was defined as fungal and bacterial taxa present in at least 75% of all samples. The core microbiome of apple fruit accounted for 13.6% of the bacterial and 63.4% of the fungal communities across all the investigated locations. We found two bacterial genera, namely *Sphingomonas* and *Methylobacterium* and six fungal genera i.e. *Aureobasidium*, *Cladosporium*, *Alternaria*, *Filobasidium*, *Vishniacozyma and Sporobolomyces*. This is a considerably low number of taxa, relative to other reported core microbiomes in plants (Pfeiffer *et al*., 2016). However, this can be attributed to the high number of samples in the present study; which lowers the probability that same taxon will be present in all samples and the evaluation of samples from different countries and tissue‐types.

*Sphingomonas*, a gram‐negative, non‐motile, aerobic bacterial genus, is known for its bioremediation of heavy metals and biodegradation of polycyclic aromatic hydrocarbons, and is associated with plant growth promotion through its ability to produce gibberellins and indole acetic acid in response to different abiotic stress conditions, such as drought, salinity and heavy metal stresses (Asaf *et al*., 2020). Interestingly, those phytohormones are also involved in fruit maturation, development and quality. For example, fruit‐set in tomato (*Solanum lycopersicum*) depends on gibberellins and auxins (Serrani *et al*., 2010; Liu *et al*., 2018b). Similarly, *Methylobacterium* is a gram‐negative, aerobic, motile bacterial genus with plant growth‐promoting properties (Krug *et al*., 2020). *Sphingomonas* and *Methylobacterium* have been previously reported as a component of the apple microbiome and as two of their predominate genera (Liu *et al*., 2018a; Wassermann *et al*., 2019a; Wassermann *et al*., 2019b; Abdelfattah *et al*., 2020), as well as a component of the core microbiome in several other plant species (Pirttilä *et al*., 2000; Delmotte *et al*., 2009; Mezzasalma *et al*., 2018; Trivedi *et al*., 2020). *Aureobasidium* and *Cladosporium* have also been reported as a common member of the microbiome of apple (Abdelfattah *et al*., 2016; Wassermann *et al*., 2019b) and other plants (Abdelfattah *et al*., 2015; Abdelfattah *et al*., 2018b; Abdelfattah *et al*., 2019). These taxa can be found as endophytes or epiphytes in association with various plant organs, e.g., leaves, flowers, fruit, seed and so forth. Although the core microbiome is typically considered to have a high level of specificity between species, the common reporting of these taxa suggests the possibility of a core microbiome that is shared between different plant species. This commonality is expected to exist at the level of genus and that some degree of species specificity may exist. In this regard, we found that the core bacterial genera, *Sphingomonas* and *Methylobacterium* accounted for a considerable fraction of the observed variation between the investigated locations, as well as tissue types. For example, the community composition of either *Sphingomonas* or *Methylobacterium* was sufficient to distinguish between most of the investigated countries. Similar results were also observed for *Aureobasidium*, a core fungal genus, however, species variability was limited, and differences were attributed to niche specialization in the different tissue‐types. Notably, both bacterial genera appeared to be distinct in tissue types. The geographical location demonstrated to be an important determinants of the *Methylobacterium* community composition in the plant phyllosphere *(*Knief *et al*., *2010* *)*. The presence of distinct *Sphingomonas* community in different fruit tissue‐types suggests site‐specialization to these microhabitats. Interestingly, the majority of the fungal core microbiome was represented by yeasts with known antagonistic activity against pre‐ and postharvest pathogens. Despite being one of the most common fungi associated with apples, *Penicillium*, the causal agent of the most important apple postharvest disease, blue mould (Vero *et al*., 2002; Hocking, 2014; Ballester *et al*., 2017), was not found to be a component of the core microbiome. *Penicillium* species are able to grow and proliferate at low temperatures during cold storage, giving them an advantage over other fungal species (Abdelfattah *et al*., 2020). In this regard and considering samples in the present study were collected immediately after harvest, it can explain the low prevalence and the absence of *Penicillium* from the apple core microbiome. Information about the core microbiome can be further used to develop biological control strategies against apple diseases and disorders. Though core species, by definition, are detected across all samples, their relative abundance pattern vary and, in some cases, forms characteristic groups of microorganisms. Dissecting the microbiome into co‐occurrence modules can serve the construction of synthetic communities with distinct function (Vannier *et al*., 2019). For example, such associations can serve the design of multiple‐species synthetic communities for achieving an efficient biocontrol activity. Alternatively, dissecting the microbiome into microbial modules can allow limiting the search for a single efficient antagonist agent. In the context of the apple fruit microbiome, co‐occurrence patterns have stratified the fruit microbiome into five key groups with core genera located in two of them: one cluster with *Aureobasidium*, and the second with *Cladosporium*, the two most abundant Ascomycota genera. Though most of the significant interactions detected in the network are positive, some negative associations allow formulating predictions for potential biocontrol agents against pathogens. Based on the network view, experimental design of potential biocontrol agent could compare the activity of a single microorganism versus consortium representing a native co‐occurring module. Potential biocontrol strategies can hence benefit from the network view of microbiome interactions allow to go beyond the single biocontrol agent to the educated design of a biocontrol consortium.

## Conclusions

Recent studies have demonstrated the critical role that the plant microbiome plays in plant health, fitness and productivity. Less attention, however, has been given to studies on the carposphere, compared with the rhizosphere and phyllosphere. Apple fruit were recently reported to host a high microbial diversity with niche specialization exhibited in calyx‐end, stem‐end and peel tissues. Whether this niche specialization is consistent in different apple‐production areas globally and whether a ‘core’ microbiome exists, regardless of geographic location, as has been reported for the rhizosphere of other fruit crops has not been determined. In the present study, the microbial communities associated with ‘Royal Gala’ apple were characterized using amplicon‐based high‐throughput sequencing to assess both large‐ and small‐scale spatial variations and to determine the presence of a core microbiome and hub microbes. Such information is critical for understanding the role of microbiome in the susceptibility of apple fruit to pre‐ and postharvest diseases, fruit safety and potentially fruit quality during long‐term storage.

Here, we demonstrated that the microbiome of the apple fruit collected from similar climates, within a continent or hemisphere, exhibiting the highest degree of similarity. Notably, fungal communities were more variable than bacterial communities in terms of diversity and abundance. In addition, we showed that the distinct composition of the different tissue‐types is a global feature of the apple microbiome. Six fungal genera (*Aureobasidium*, *Cladosporium*, *Alternaria*, *Filobasidium*, *Vishniacozyma* and *Sporobolomyces*) and two bacterial genera (*Sphingomonas* and *Methylobacterium*) were defined as representing the core microbiome. Overall, the findings in the present study may suggest local adaptations of the apple microbiome to local environment. Regarding the spatial variations within the fruit, we suggest for future apple microbiome studies to consider these variations during their experimental design and sampling strategies by either analysing different fruit tissues separately or including the whole fruit to minimize discrepancies between studies. In addition, it would be interesting for future fruit microbiome works to investigate whether the variations among fruit tissue types can be generalized to all fruits.

## Experimental procedures

‘Royal Gala’ apple fruit harvested at commercial maturity were used in this study. Fruit were harvested in four regions (North America, South America, Europe and the Middle East) that included 21 locations in eight countries (USA, Canada, Uruguay, Italy, Spain, Switzerland, Israel and Turkey). Fruit were harvested at commercial maturity using standard maturity indices. Harvesting occurred in July–September in the northern hemisphere and February–March in the southern hemisphere (Table S1). A standardized protocol was used for sample collection and processing in all sampling locations across countries, then the extracted DNA was sent USDA‐ARS, WV, USA, to avoid bias introduced by library preparation and sequencing. Briefly, in each locations/orchard, eight trees (not adjacent to each other) were selected and five fruit/tree were sampled from around the circumference of the tree. Each tree consisted one replicate; total of eight replicates per location/orchard. Five fruit from each tree are pooled to make one biological replicate (total eight biological replicates/orchard). From each apple, three tissue types (peel, stem‐end and calyx‐end) were sampled as previously described (Abdelfattah *et al*., 2016). First, a sterile cork‐borer was used to excise the fruit core and the top and bottom 1.5 cm were used as stem‐ and calyx‐end, respectively. To collect the peel, a thin layer around the fruit equator with approximately 1.5 cm in width was obtained from each apple using a peeler. Samples from of the same fruit tissue from the same tree were pooled and considered a biological replicate making total of eight replicate of each tissue site per orchard and a total of 505 samples globally. Samples were immediately frozen in liquid nitrogen, kept in −80 °C until freeze‐dried.

### Libraries and sequencing, data processing, downstream and statistical analysis

Lyophilized samples were homogenized, and their DNA was extracted using DNeasy PowerLyzer PowerSoil Kit (Qiagen, Germantown, MD, USA). Initial tissue disruption of 250 mg was performed with a Qiagen PowerLyzer 24 Homogenizer (Qiagen, Germantown, MD, USA). DNA extractions were automated using a Qiagen QiaCube (Qiagen, Germantown, MS, USA), using the processing routine recommended by the manufacturer for the PowerSoil kit. Extracted DNA was used as the template for amplicon PCR reactions that amplified the bacterial 16S ribosomal region and the fungal internal transcribed spacer (ITS) region. The V4 region of 16S rRNA was amplified using the universal primers 515F (Parada *et al*., 2016) and 806R (Apprill *et al*., 2015) in conjunction with peptide nucleic acids (PNAs) (PNA Bio) added to inhibit amplification of ribosomal and mitochondrial sequences (Lundberg *et al*., 2013). ITS amplicons were amplified using ITS3/KYO2 (Toju *et al*., 2012) and ITS4 (White *et al*., 1990) primers along with a custom‐designed blocking oligo designed to inhibit amplification of the host DNA (5′ ATTGATATGCTTAAATTCAGCGGGTAACCCCGCCTGACCTGGGGTCGCGTT‐C3 spacer 3′). All primers were modified to include the necessary Illumina adapters (www.illumina.com) for subsequent PCR addition of Illumina indexes for multiplexing.

For bacteria, PCR reactions were conducted in a total volume of 25 μl containing 12.5 μl of KAPA HiFi HotStart ReadyMix (Kapa Biosystems), 1.0 μl of each primer (10 μM), 2.5 ul of mitochondrial PNA (5 uM), 2.5 μl of plastid PNA (5 uM), 2.5 μl of DNA template and 3 μl nuclease‐free water. Reactions were incubated in a T100 thermal cycler (BioRad) at 95°C for 5 min followed by 30 cycles of 95°C for 30 s, 78°C for 5 s, 55°C for 30 s, 72°C for 30 s and a final extension at 72°C for 5 min. For fungal (ITS) amplicon generation, 25 μl PCR reactions contained 12.5 μl of KAPA HiFi HotStart ReadyMix (Kapa Biosystems), 1.0 μl of each primer (10 μM), 1.0 μl of blocking oligo (10 uM), 2.5 μl of DNA template and 7 μl nuclease‐free water. Reactions were incubated in a T100 thermal cycler (BioRad) at 95°C for 5 min followed by 30 cycles of 95°C for 30 s, 55°C for 30 s, 72°C for 30 s a final extension at 72°C for 5 min. Library preparation following amplicon PCR was performed as specified in the Illumina 16S Metagenomic Sequencing Library Preparation guide precisely as outlined. Subsequent library size, quality and confirmation of the absence of adapter dimers were performed on an Agilent 2100 Bioanalyzer (Agilent). Paired‐end sequencing of amplicons was done on an Illumina MiSeq (Illumina) sequencer with a V3 600‐cycle Reagent Kit (Illumina).

### Data analysis

Demultiplexing, merging forward and reverse reads, quality filtering and trimming and ASVs generation were done using DADA2 as integrated in Qiime2 ADDIN EN.CITE (Callahan *et al*., 2016; Bolyen *et al*., 2019). Taxonomic assignment of ASVs was done using a similarity threshold of 97% against the Greengenes database for 16S reads and against the UNITE database for ITS reads (Abarenkov *et al*., 2010; DeSantis *et al*., 2006). Plant related sequences e.g. chloroplast and mitochondria, were filtered out before the following analyses. Rarefaction to an even sequencing depth of 1000 reads per sample was used to normalize ITS and 300 reads for the 16S features tables which were used to calculate Shannon diversity. To evaluate the effect of the geographical location, orchard and tissue type (i.e., stem‐end, calyx‐end and peel) on the fungal and bacterial diversity, Analysis of Variance Model (a wrapper for fitting linear models) was used. Here, we modelled Shannon index as a response variable and country, orchard, and tissue as fixed effects. In addition, pairwise comparisons between Shannon index of the fungal and bacterial diversity among the sampling locations (countries) was done using Wilcox test and the p values were corrected using FDR method. For the subsequent analysis, the unrarefied ASV table was normalized using MetagenomeSeq's Cumulative Sum Scaling (CSS; Paulson *et al*., 2013). Differences in community composition between the investigated countries, orchards and tissue types were tested using Permutational Multivariate Analysis of Variance Using adonis (~PERMANOVA) in vegan R with 999 permutations. Here, we modelled the distance among countries in their community composition, based on Bray–Curtis dissimilarity index as a response variable and country, orchard and tissue as fixed effects. Hierarchical clustering of the community composition based on Bray–Curtis dissimilarity distances with ‘average’ as the clustering method was performed using *hclust* in R Package ‘*stats*” version 4.0.1 (Oksanen *et al*., 2007). These results were visualized in dendrograms using *fviz_dend* function in the R package factoextra version 1.0.7 (Kassambara and Mundt, 2020).

The core microbiome was calculated based on genera present in at least 75% of the investigated samples using *core* function in *Microbiome* package (Salonen *et al*., 2012). Interactions between core and non‐core species were limited to genera whose normalized relative abundance >0.1% (average across replicas) in at least a single sample. Co‐occurrences were described based on Spearman's rho correlation coefficients between the distribution patterns of the genera joining the normalized bacterial and fungal tables. Scores were calculated using ‘Pandas.corr’ python package v1.1.0. Correlation matrix and visualized using ‘seaborn.clustermap’ python package v0.10.1. Co‐occurrence networks were generated based on correlation scores. Network visualization and the positioning of the nodes were calculated according to the force‐directed Fruchterman & Reingold algorithm used for calculating layouts of simple undirected graphs (Buchfink *et al*., 2015). The algorithm was implemented using nx.draw function via the ‘pos’ parameter in the ‘NetworkX’ python package v1.11. Node degree was calculated using the nx.degree function. Visualization was generated using ‘Plotly’ python package v4.9.0. Linear discriminant analysis effect size (LEfSe; Segata *et al*., 2011) was used for biomarker discovery to determine a list of taxa that best characterize each geographical location (Country). Higher LEfSe score indicate higher consistency of differences in relative abundance between taxa of each country.

## Author contributions

S.D and M.W. conceptualized and designed the experiments. Y.V.Z., A.K., A.B. S.S., O.F., E.B. performed the experiments; A.A, S.F., R.B. analysed the data. C.D., J.L., A.K., W.E., S.A., D.S., R.T., N.T., O.O., A.B., S.V. P.D. sampled the fruit in different countries and extracted DNA from fruit tissues. A.A. wrote the first draft and M.W. and S.D. made a major contribution to the final version. S.D. and M.W. supervision and project administration. G.B. analysis of the data and critically read the manuscript. All authors have read and agreed to the published version of the manuscript.

## Supporting information


**Fig. S1**. Hierarchical clustering showing the similarity among apple fungal (A) and bacterial (B) communities composition collected from different countries, i.e. Canada, Turkey, Israel, Italy, Uruguay, USA West, USA East, Switzerland and Spain.

**Fig. S2**. A) a phylogenetic tree of the most prevalent Sphingomonas ASVs which were at least present with 0.1%. B) hierarchical clustering of Sphingomonas community. C) PCA ordination showing the variation in Sphingomonas (the core genus) community between fruit tissue types in all investigated orchards.

**Fig. S3**. Distribution of node‐degree of core and none‐core species in co‐occurrence network. Most core species have multiple links (>13). Node degree of core species is significantly higher than non‐core species (Wilcoxon *P* = 0.0039).

**Table S1**. A list summarizing the information about the apple samples included in the present study.

**Table S2**. Taxonomy and relative abundance of the most prevalent fungal and bacterial taxa detected on apple fruit in each of the investigated countries (Canada, Turkey, Israel, Italy, Uruguay, USA West, USA East, Switzerland and Spain).

**Table S3**. Pairwise comparisons of the fungal and bacterial diversity (based on Shannon index) between the sampling locations (Canada, Turkey, Israel, Italy, Uruguay, USA West, USA East, Switzerland and Spain) using Wilcox test and corrected using FDR method. *P* values less than 0.05 were considered significant.

## Data Availability

The datasets generated and/or analysed during the current study are available in the [SRA NCBI] repository, and can be accessed from the following link https://www.ncbi.nlm.nih.gov/bioproject/702262.
