# Supplementary figures and images for: Global analysis of the apple fruit microbiome: are all apples the same?
Source: Environ Microbiol. 2021 Apr 1;23(10):6038–55. doi: 10.1111/1462-2920.15469 (PMC8596679; doi:10.1111/1462-2920.15469)

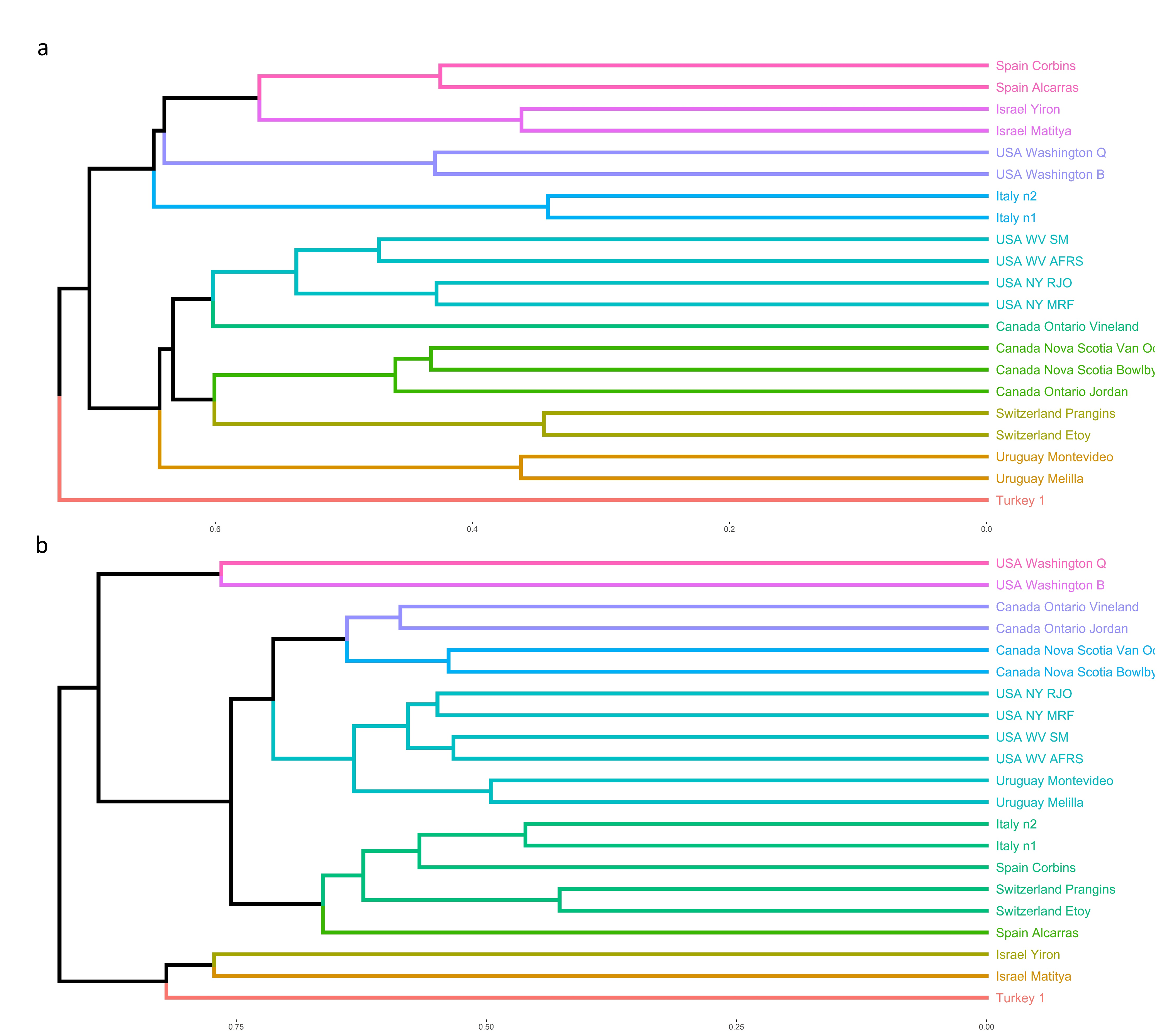

Supplement: Supplementary file 1 — Fig. S1. Hierarchical clustering showing the similarity among apple fungal (A) and bacterial (B) communities composition collected from different countries, i.e. Canada, Turkey, Israel, Italy, Uruguay, USA West, USA East, Switzerland and Spain. [file EMI-23-6038-s005.tif]

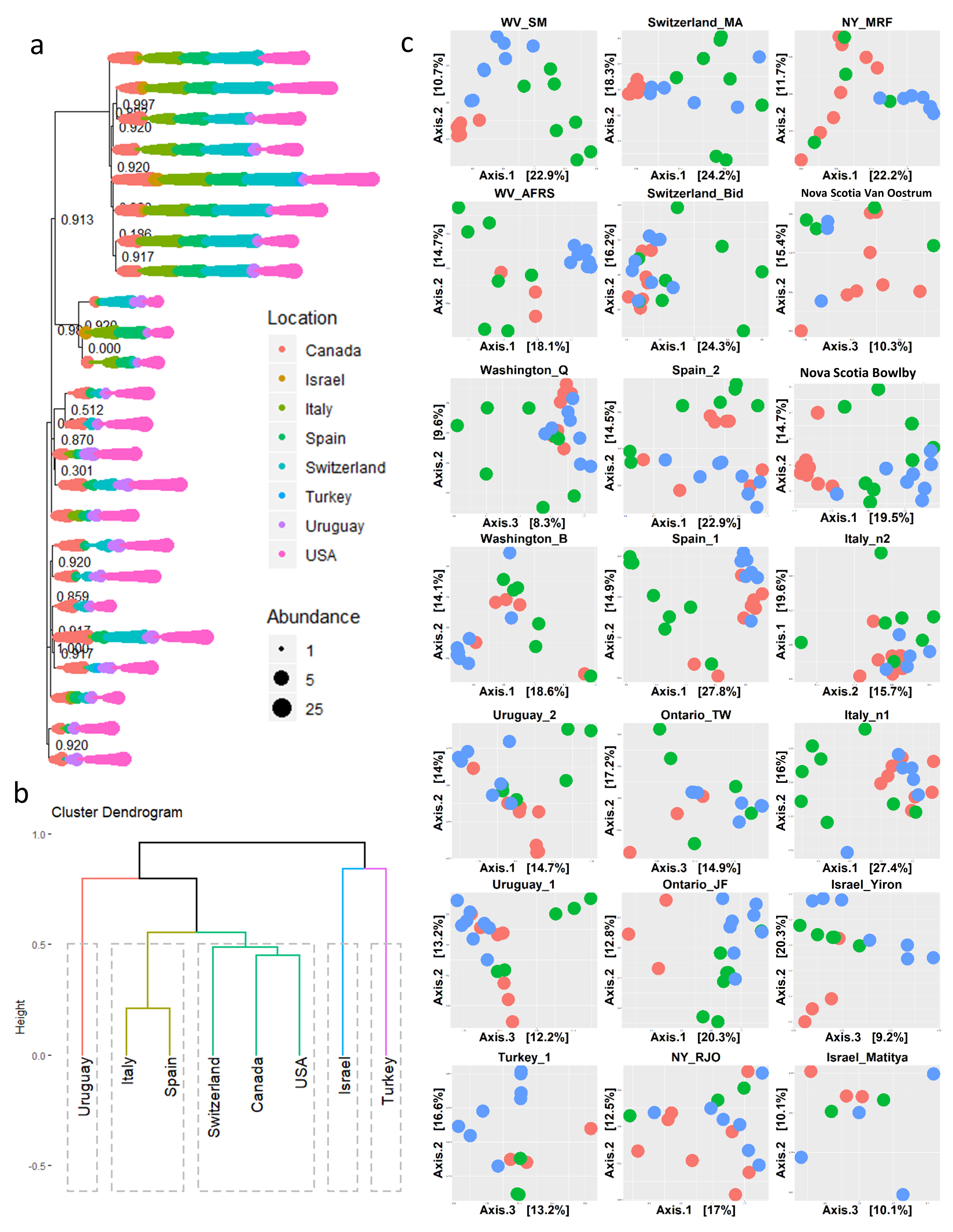

Supplement: Supplementary file 2 — Fig. S2. A) a phylogenetic tree of the most prevalent Sphingomonas ASVs which were at least present with 0.1%. B) hierarchical clustering of Sphingomonas community. C) PCA ordination showing the variation in Sphingomonas (the core genus) community between fruit tissue types in all investigated orchards. [file EMI-23-6038-s004.tif]

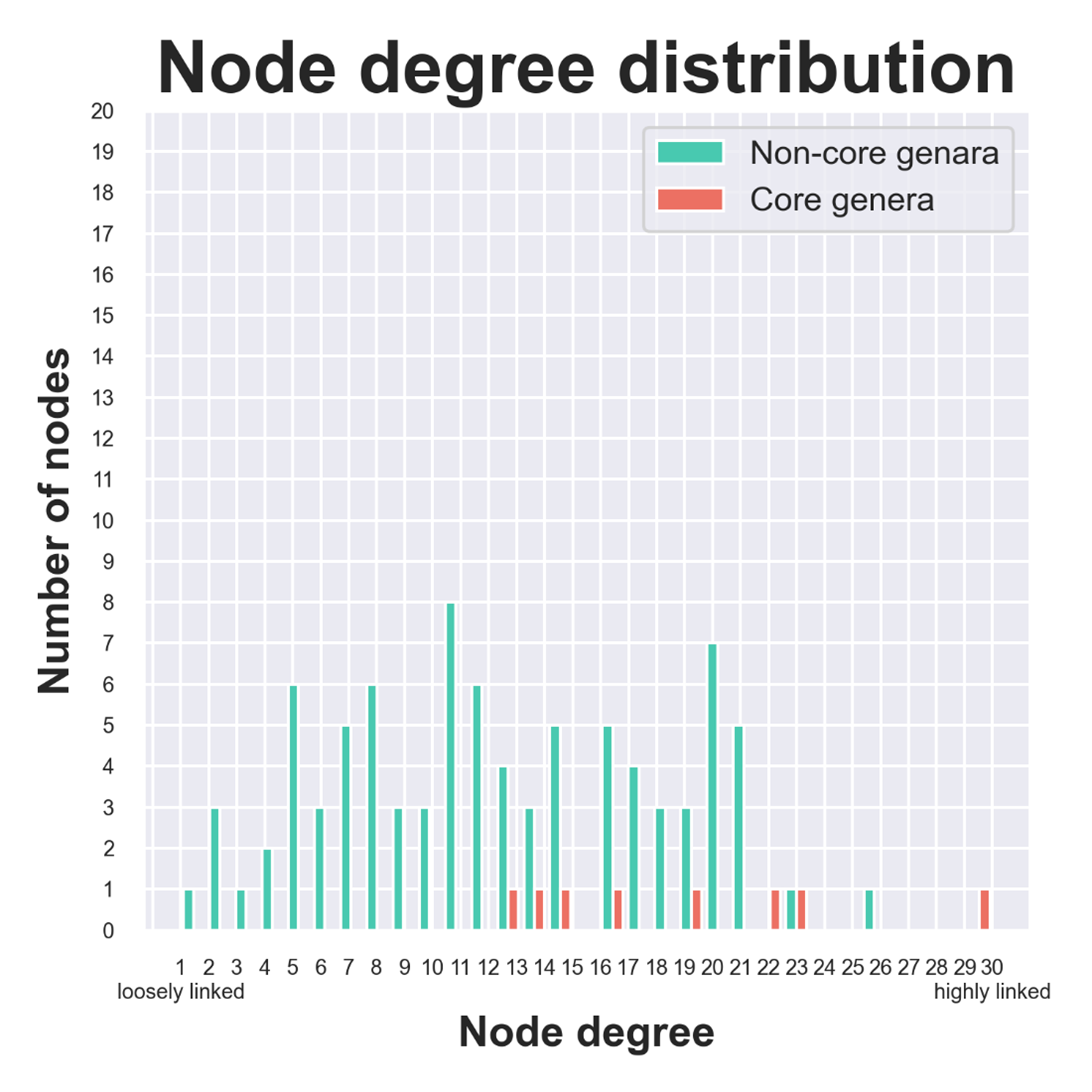

Supplement: Supplementary file 3 — Fig. S3. Distribution of node‐degree of core and none‐core species in co‐occurrence network. Most core species have multiple links (>13). Node degree of core species is significantly higher than non‐core species (Wilcoxon P = 0.0039). [file EMI-23-6038-s006.tif]
