# Supplementary material for: Global analysis of the apple fruit microbiome: are all apples the same?
Source: Environ Microbiol. 2021 Apr 1;23(10):6038–55. doi: 10.1111/1462-2920.15469 (PMC8596679; doi:10.1111/1462-2920.15469)
Supplement: Supplementary file 6 — Table S3. Pairwise comparisons of the fungal and bacterial diversity (based on Shannon index) between the sampling locations (Canada, Turkey, Israel, Italy, Uruguay, USA West, USA East, Switzerland and Spain) using Wilcox test and corrected using FDR method. P values less than 0.05 were considered significant. [file EMI-23-6038-s001.docx]

| **Shannon** | **Pairwise Wilcox test** | **Canada** | **Israel** | **Italy** | **Spain** | **Switzerland** | **Turkey** | **Uruguay** | **USA East** |
| --- | --- | --- | --- | --- | --- | --- | --- | --- | --- |
| **Fungi** | Israel | 6.3e-05 | - | - | - | - | - | - | - |
|  | Italy | 2.8e-09 | 3.4e-05 | - | - | - | - | - | - |
|  | Spain | 0.00025 | 0.94979 | 1.7e-05 | - | - | - | - | - |
|  | Switzerland | 0.76827 | 7.7e-08 | 6.1e-14 | 4.8e-06 | - | - | - | - |
|  | Turkey | 6.2e-05 | 0.00897 | 0.76827 | 0.00230 | 3.6e-05 | - | - | - |
|  | Uruguay | 0.39254 | 1.1e-07 | 6.1e-14 | 2.9e-07 | 0.27280 | 1.1e-05 | - | - |
|  | USA East | 3.6e-05 | 0.71225 | 4.8e-05 | 0.89052 | 3.7e-06 | 0.01697 | 2.6e-07 | - |
|  | USA West | 0.51518 | 1.3e-07 | 5.5e-12 | 4.8e-06 | 0.27679 | 2.8e-05 | 0.76524 | 1.0e-06 |
| **Bacteria** | Israel | 0.00381 | - | - | - | - | - | - | - |
|  | Italy | 6.1e-12 | 2.5e-06 | - | - | - | - | - | - |
|  | Spain | 0.00053 | 0.61454 | 9.0e-06 | - | - | - | - | - |
|  | Switzerland | 1.8e-09 | 0.00018 | 0.21425 | 0.00079 | - | - | - | - |
|  | Turkey | 0.71092 | 0.04159 | 1.1e-06 | 0.01809 | 0.00011 | - | - | - |
|  | Uruguay | 0.01366 | 0.94239 | 3.0e-05 | 0.61454 | 0.00085 | 0.06020 | - | - |
|  | USA East | 0.00077 | 0.61454 | 0.00053 | 0.85420 | 0.01350 | 0.02217 | 0.53031 | - |
|  | USA West | 4.8e-05 | 0.12317 | 0.00297 | 0.28354 | 0.08451 | 0.00338 | 0.19399 | 0.52344 |

Table S3 Pairwise comparisons between the fungal and bacterial diversity (Shannon) sampling locations (countries) using Wilcox test.
